# Physicochemical and Digestion Properties of Potato Starch Were Modified by Complexing with Grape Seed Proanthocyanidins

**DOI:** 10.3390/molecules25051123

**Published:** 2020-03-03

**Authors:** Zirui Zhang, Jinhu Tian, Haitian Fang, Huiling Zhang, Xiangli Kong, Dongmei Wu, Jiaqi Zheng, Donghong Liu, Xingqian Ye, Shiguo Chen

**Affiliations:** 1 National-Local Joint Engineering Laboratory of Intelligent Food Technology and Equipment, Zhejiang Key Laboratory for Agro-Food Processing, Zhejiang Engineering Laboratory of Food Technology and Equipment, Fuli Institute of Food Science, College of Biosystems Engineering and Food Science, Zhejiang University, Hangzhou 310058, Zhejiang, China; zirui_zhang@zju.edu.cn (Z.Z.); 11813017@zju.edu.cn (D.W.); jiaqizheng789@126.com (J.Z.); dhliu@zju.edu.cn (D.L.); psu@zju.edu.cn (X.Y.); 2 Ningxia Key Laboratory for Food Microbial-Applications Technology and Safety Control, Ningxia University, Yinchuan 750021, Ningxia, China; fanght@nxu.edu.cn (H.F.); zhl5792@163.com (H.Z.); 3 Institute of Nuclear Agricultural Sciences, Zhejiang University, Hangzhou 310058, Zhejiang, China; kongxiangli1980@hotmail.com; 4 Ningbo Research Institute, Zhejiang University, Ningbo 315100, Zhejiang, China

**Keywords:** proanthocyanidins, potato starch, complex, physicochemical properties, in vitro digestion

## Abstract

Dietary intake of potato starch could induce a dramatic increase in blood glucose and is positively associated with chronic metabolic diseases (type II diabetes, cardiovascular disease, etc.). Grape seed proanthocyanidins (GSP) are known to decrease starch digestion by inhibiting digestive enzymes or changing the physicochemical properties of starch. In the present study, GSP were complexed with potato starch to prepare polyphenol–starch complexes. The physiochemical properties and digestibility of complexes were investigated by in vitro digestion model, X-ray diffraction, differential scanning calorimetry, rapid visco analyzer, Fourier transform infrared spectroscopy as well as texture profile analysis. Results indicated that the peak viscosity, breakdown, trough, and setback of the complexes disappeared, replaced by a special continuous increase in paste viscosity. The complexes showed a lower final viscosity and higher thermal stability with the increasing binding amount of GSP. GSP decreased the hardness of the complexes’ gel significantly. FT-IR indicated that GSP might interact with potato starch through noncovalent forces. Additionally, the complexes also showed a higher content of slowly digestible starch and resistant starch than that of the native starch. Thus, we inferred that the addition of GSP could modify the digestibility of potato starch and be an optional way to modify the starch with lower digestion.

## 1. Introduction

Potato (*Solanum tubersum* L.) is an important carbohydrate crop that is widely planted around the world [1]. In 2017, the production of potato was 388 million tons worldwide and is expected to increase in the future [2]. However, due to the high content of rapidly digestible starch (RDS), potato has been classified into middle or high glycemic index (GI) foods, which would induce a dramatic change in blood glucose [3]. There is wide consideration that long term consumption of high GI food would break the balance of blood glucose and give rise to chronic diseases such as obesity, fatty liver, hyperglycemia, and type II diabetes [4]. Thus, ways to reduce starch digestion will be urgently needed.

Proanthocyanidins are polyphenol compounds of flavanol monomers together with their polymers, which are the secondary metabolites of plants [5]. Grape seed, as a by-product of the grape juice/wine industry, contains an abundance of polyphenols, especially proanthocyanidins, which show potential anti-oxidant, anti-bacterial, and anti-diabetic properties [6]. Particularly, proanthocyanidins have attracted more and more attention in the regulation of starch digestion [7,8]. On one hand, proanthocyanidins could bind to the amino acid residues of digestive enzymes such as α-amylase/α-glucosidase and inhibit their activities, thus reducing the amount of glucose released from starch during digestion [9,10,11]. On the other hand, proanthocyanins could occupy the helical structure of starch through hydrophobic interactions, or its hydroxyl and carbonyl groups could bond with the hydroxyl groups of starch by hydrogen bonding, and change the physicochemical properties as well as the digestibility of starch [8,12].

The digestion control properties have stimulated research interest and resulted in the production of complexes with different methods. However, the formation mechanism of the complex could be mainly divided into two ways. First, after treatment (alkali solution [13]; high temperature [13]; enzymatic action [14]), amylose forms a left-hand spiral cavity [15]. Polyphenols could enter the cavity of the amylose helix and form an inclusion complex through hydrophobic interaction [16]. Second, considering the bulky size and lack of hydrophobicity of some polyphenols such as grape seed proanthocyanidins, and the limited size of the cavity, these polyphenols could not form inclusion complexes with starches. Regarding that both polyphenols and starch molecules are rich in hydroxyl groups, the interaction is mainly through hydron bonding [12]. The formation of complexes also greatly affects the physicochemical and nutritional properties of starches [17]. The effects on starch properties and the mechanism of interaction are complex because of the difference in the type of phenolic compounds and starches, and the methods of preparation. To the best of our knowledge, few studies have paid attention to the physicochemical and digestion properties of the complexes formed by potato starch and grape seed proanthocyanidins (GSP).

In the present study, GSP–potato starch complexes were prepared in aqueous ethanol above the pasting temperature of potato starch. The binding quality, physicochemical, and nutritional properties were also investigated. We hypothesized that the complexes between potato starch and GSP might be stabilized by noncovalent bonds, especially by hydrogen bonding. Therefore, the purpose of this study was to reveal the impact of GSP on the physiochemical properties and digestibility of potato starch. The understanding of interactions between GSP and potato starch might facilitate the reuse of grape processing by-products, provide a new idea to the application of GSP in novel functional starchy food, and help others have a better understanding of the reaction between proanthocyanidins and starches.

## 2. Results

### 2.1. Binding Ability of GSP with Potato Starch

As shown in Table 1, the loading efficiency of GSP ranged from 68.49% to 73.41%, which indicated a relatively high affinity between potato starch granules and GSP. The binding amount was positively correlated with the GSP concentration (7.36 to 35.72 mg/g starch from 1% to 5% GSP addition). Before gelatinization, the integrity of starch granules could limit the access between guest molecules and starch amylose/amylopectin, thus starch only had a lower binding amount [18]. When the GSP were heated with potato starch at 70 °C, the breakdown of potato starch granule and unwinding of the starch double helix structure would make it much easier for GSP to access the inside and increase the binding amount [12].

### 2.2. Semi-Crystalline Character of Potato Starch with GSP

**Figure 1:**
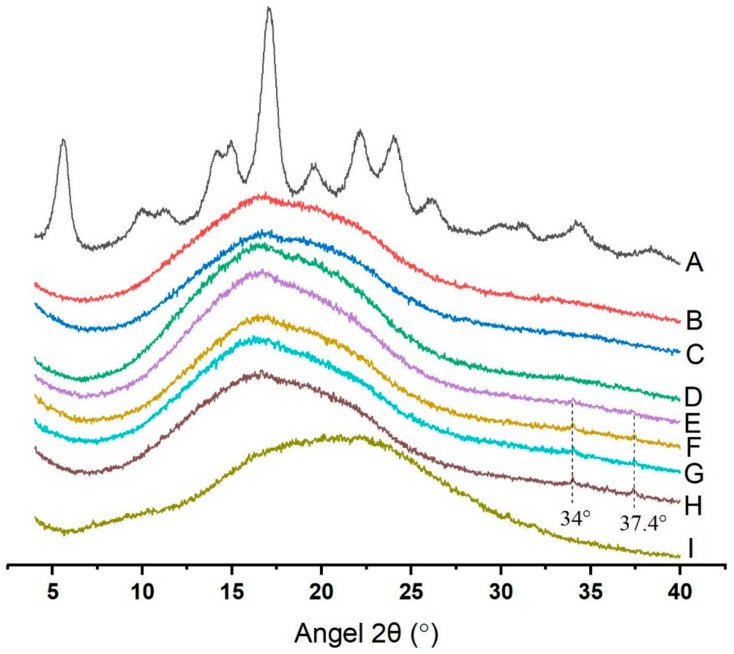
X-ray diffraction analysis of the native starch, GSP–potato starch complexes and GSP. (A) Native starch; (B) Starch + 0.0% GSP; (C) Starch + 1.0% GSP; (D) Starch + 2.0% GSP; (E) Starch + 3.0% GSP; (F) Starch + 4.0% GSP; (G) Starch + 4.5% GSP; (H) Starch + 5.0% GSP; (I) GSP.

As shown in Figure 1, diffraction peaks of native potato starch were observed at 2θ of 5.7°, 14.9°, 17°, and 24°, which was confirmed to be a typical B-type pattern [19]. No clear peaks in GSP-potato starch complexes were observed, indicating that little crystalline region existed in those samples. In other words, the absence of clear peaks in the diffractograms indicated that the long-range crystalline structure of potato starch had been destroyed [20]. However, two tiny new peaks were noticed at approximately 2θ of 34° and 37.4° in Starch + 3.0% GSP, Starch + 4.0% GSP, Starch + 4.5% GSP, and Starch + 5.0% GSP. Considering both raw potato starch and GSP showed no peak at those positions, we inferred that the interaction between GSP and potato starch might form a new crystal structure. A similar result has also been reported where a new peak at 30° appeared in the diffraction diagram of Lotus leaf flavonoid–starch complexes [21].

### 2.3. Pasting Properties of GSP–Potato Starch Complexes

**Figure 2:**
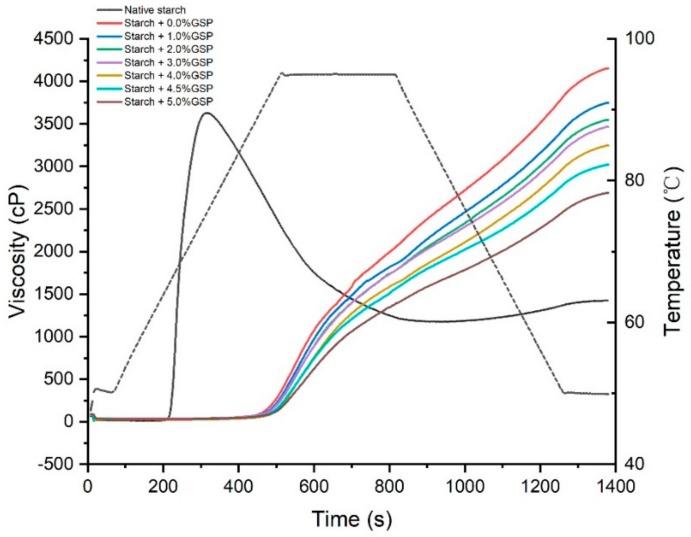
Pasting properties of native starch and GSP–potato starch complexes with different binding amounts of GSP.

The pasting properties of native potato starch and GSP-potato starch complexes are shown in Figure 2 and Table 2. The native potato starch showed a normal pasting profile of potato starch [22]. However, a significant increase in pasting temperature and a continuously increasing viscosity were observed in GSP–potato starch complexes during the whole RVA testing, regardless of the GSP concentration. This result indicated that the complexes were very stable under the conditions of high temperature and continuous shearing [22]. Considering the destruction of the complexes semi-crystallinity, the special RVA patterns could be attributed to the enhancement of ordering of amorphous regions in starch granules after treating with restricted temperature and water content. Hence, the complexes need more energy for the disruption of their structure and the formation of paste [23], resulting in the higher pasting temperature and the continuously increasing viscosity.

When the concentration of GSP increased from 0.0% to 5.0%, the final viscosity decreased from 4119.0 to 2623.0 cP and the gelatinization temperature further increased from 91.9 °C to 95.1 °C. During the preparation of the complexes, gelatinization unwinds the starch double helix and results in the loss of the crystalline region, then GSP migrates into the starch granule and intrudes into the amorphous region [24]. The unordered starch molecules and GSP may interact with each other by hydrogen bonding, and impede the rearrangement of amylose and amylopectin [12]. We speculate that when the complexes were heated in the RVA apparatus, GSP was released from potato starch, and competed with starch for binding water molecules, and thus prevented the swelling of starch and resulted in lower final viscosity and higher gelatinization temperature. The lower viscosity and the disappearance of breakdown in complexes implied a better heat and shear stability, which might be a good ingredient in the industry of noodles.

### 2.4. Thermal Properties of GSP–Potato Starch Complexes

The thermal properties of the native potato starch and complexes were shown in Table 3. Compared with native potato starch, the To, Tp, Tc, and ΔH values of Starch + 0.0% GSP showed a significant decrease, indicating that pre-gelatinizing and lyophilizing lowered the thermostability of the starch [25]. However, compared with Starch + 0.0% GSP, To, Tp, and Tc of the complexes showed an upward trend with the increased binding of GSP. It has been reported that the thermal properties were influenced by the degree of starch crystallinity [26]. The To value represents the melting temperature of the most unstable crystallites, while the Tp and Tc values represent the majority and most stable crystallites in starch [27]. Hence, the slight change in To, Tp, and Tc values might indicate that GSP changed the crystalline structure in potato starch. In the present study, the enthalpy value (ΔH) increased significantly from 0.9 to 1.5 J/g as the GSP binding amount increased. Similar results were also found by Oladele et al. [5], who also reported a significantly increased ΔH for grape pomace extract modified maize starch. The strong interaction between phenolic compounds and starch might exist in the complex, which needed more energy to break the structure.

### 2.5. FT-IR Analysis

**Figure 3:**
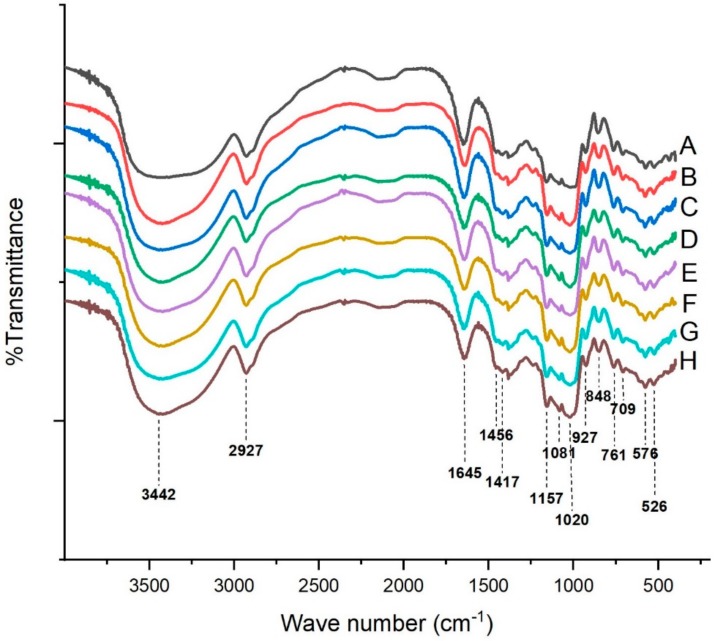
Fourier transform infrared analysis of the native starch and GSP-potato starch complexes. (A) Native starch; (B) Starch + 0.0% GSP; (C) Starch + 1.0% GSP; (D) Starch + 2.0% GSP; (E) Starch + 3.0% GSP; (F) Starch + 4.0% GSP; (G) Starch + 4.5% GSP; (H) Starch + 5.0% GSP.

The FT-IR spectra of the native starch and complexes are shown in Figure 3. As shown, the infrared band between 3500 and 3100 cm^−1^ showed a broadband, which belonged to the stretch vibration of O–H groups and absorption of H-bonding. The 2927 cm^−1^ belonged to the anti-symmetrical stretching vibration of CH_2_. The band at 1645 cm^−1^ was assigned to the absorption of the amorphous region in starch [28]. The band at 1456 cm^−1^ referred to the bending vibration of CH_2_, while 1417 cm^−1^ referred to the C–O–O stretching vibration [28]. The other absorption peaks in the fingerprint region between 1200 and 670 cm^−1^ were 1157 cm^−1^ (C–C, C–O stretching), 1080 and 1020 cm^−1^ (both due to C–O stretching), 927 cm^−1^ (skeletal mode vibrations of α-1,4 glycosidic bond linkage), 848 cm^−1^ (CH2 wagging vibration), 761 cm^−1^ (C–C stretching), 709 cm^−1^ (C–C stretching, skeletal modes), and 576 and 526 cm^−1^ (pyranose ring, skeletal modes) [21]. Native potato starch showed the same characteristic peaks compared with the complexes, which indicated that no covalent bonds formed between potato starch and GSP. However, the intensity of the infrared band between 3500 and 3100 cm^−1^ increased with the increase in GSP, indicating that GSP might strengthen the absorption through H-bonding. We also noticed that in Oladele’s study [5], maize starch was mixed with GPE (grape pomace extract) in 50% aqueous ethanol together with 3% NaOH and then freeze-dried to obtained starch–phenolic complexes. FT-IR showed an increase in the vibration intensity of C–O–C (1023–1018 cm^−1^) in maize starch modified with GPE, which indicated the formation of ether bonds. The formation of covalent bonds (ether bond) might be due to the ionization of starch to form an alkoxide under alkaline condition. Mathew and Abraham [29] reported the formation of ester bonds (formation of C=O peaks) between starch and ferulic acid under alkaline conditions, which was depicted by FT-IR. We should notice that the interaction between phytochemicals and starch is complex because of the different methods used to make complexes.

### 2.6. Texture Profile of the Gel

The texture profile parameters are shown in Table 4. Compared with the native potato starch, Starch + 0.0% GSP showed a significant increase in hardness, springiness, chewiness, and resilience, and a significant decrease in cohesiveness (*p* < 0.05). However, when the GSP was involved, the parameters of the texture profile showed a decreasing trend with the increasing binding amount of GSP. This indicated that after complexation with GSP, the starch gel could be much softer. The lower final viscosities might lead to lower hardness in gel formation. Cohesiveness implies the strength of the internal bonds. The hydroxyl groups on the lateral side of solubilized polyphenols might interact with starch through hydrogen bonding and prevent the starch chains from combining [24]. After gelatinizing in RVA, GSP may be released and evenly dispersed in starch gel and might interact with starch through hydrogen bonding and reduce the interactions between unwinding starch chains.

### 2.7. Digestibility of Potato Starch Complexed with GSP

**Figure 4:**
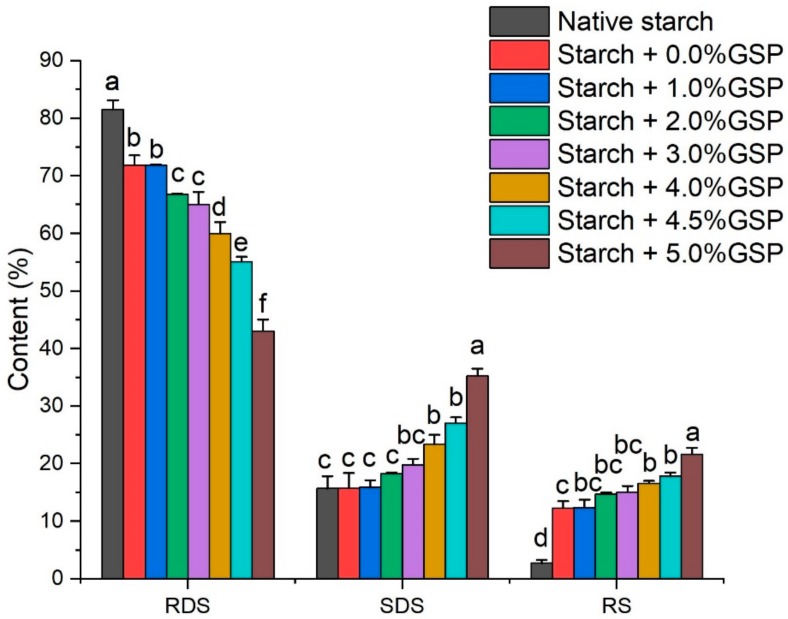
Effect of GSP–potato starch interactions on digestible properties of native starch and GSP-potato starch complexes. RDS, SDS, and RS represent rapidly digestible starch, slowly digestible starch, and resistant starch, respectively. Error bars indicate ± standard deviation. The same letters within starch digestibility types are not significantly different (*p* < 0.05).

Normally, starch is classified as rapidly digestible starch (RDS) and slowly digestible starch (SDS) and resistant starch (RS); RDS and SDS are starches hydrolyzed for 20 min and a further 100 min, RS is the starch not hydrolyzed after 120 min [30]. As shown in Figure 4, the percentage of RDS, SDS and RS in native potato starch were 81.56%, 15.71% and 2.73%, respectively. The results were consistent with previous reports [31]. RDS is generally considered to lead to a rapid increase in glucose and insulin levels in blood [32]. Thus, the high content of RSD in potato might induce a sharp fluctuation of blood glucose. In the present study, Starch + 0.0% GSP showed a significant decrease in RDS content and increase in RS content, which was 71.84% and 12.32%, respectively. However, the content of slowly digestible starch was not significantly affected, which was 15.75%. With the increasing binding of GSP, the RDS contents decreased significantly (from 71.84 to 43.07%), while SDS and RS content increased significantly (from 15.75% to 35.26% for SDS and from 12.32% to 21.67% for RS, respectively). After gelatinization, random configuration of amorphous complexes might form, which could increase space steric hindrance for amylase binding, and slow down rather than block the hydrolysis of potato starch [8]. Furthermore, GSP also had an inhibitory effect on digestion enzymes (e.g., α-amylase and α-glucosidase) [33]. Our results indicated that the addition of GSP significantly decreased the digestibility of potato starch and the increased RS could survive through the small intestine and be fermented by microorganisms in the large intestine [34].

## 3. Materials and Methods

### 3.1. Materials

GSP with a proanthocyanidin content over 95% (contains dimmer 1.82%, oligomer 76.22%), were generously provided by Tianjin Jian Feng Natural Product R&D Co. Ltd. (Tianjin, China). Epicatechin standard (SE8100) was purchased from Solarbio Science & Technology Co. Ltd. (Beijing, China). Potato starch (contains 19.40% moisture, 0.87% protein, and 1.70% fat) and amyloglucosidase (10^5^ U/mL) were bought from Aladdin Chemical Co. Ltd. (Shanghai, China). Invertase (from baker’s yeast, ≥300 U/mg) and α-amylase (porcine pancreas, 10 U/mg) were purchased from Sigma-Aldrich Co. Ltd. (St Louis, MO, USA). The total starch assay kit and the glucose oxidase/peroxidase assay kit were purchased from Megazyme International Co. Ltd. (Wicklow, Ireland). Other reagents used in the present study were of analytical grade and also purchased from Aladdin Chemical Co. Ltd. (Shanghai, China).

### 3.2. Preparation of Grape Seed Proanthocyanidins-Potato Starch Complexes

The preparation method of GSP–potato starch complexes was according to Zheng et al. [35]. Briefly, GSP in different proportions (0.0, 2.0, 3.0, 4.0, 4.5, 5.0%, based on starch weight) was first dissolved in 30% aqueous ethanol solution (prepared volume in volume). The weight of 30% aqueous ethanol solution was 10 times the weight of the starch. Then, the GSP solution was agitated with potato starch. The suspensions were incubated with magnetic stirring at 70 °C for 20 min and centrifuged to collect the supernatant and the sediments. The sediments were washed twice with 30% ethanol and then quickly frozen by liquid nitrogen and freeze-dried for 36 h to remove residual water. The sediments after drying were carefully ground with a pestle and mortar, passed through a steel 200-mesh sieve, collected, and stored at ambient temperature without light before further analysis. Complexes prepared with 0.0, 1.0, 2.0, 3.0, 4.0, 4.5, and 5.0% GSP were referred to as Starch + 0.0% GSP, Starch + 1.0% GSP, Starch + 2.0% GSP, Starch + 3.0% GSP, Starch + 4.0% GSP, Starch + 4.5% GSP, and Starch + 5.0% GSP, respectively.

### 3.3. Binding Ability of GSP with Potato Starch

The GSP that were released in the supernatant obtained in Section 3.2, which represents the free content of the GSP, were measured according to Sun et al. [36] with some modification. Briefly, 200 μL of supernatant from Section 3.2 was mixed with 500 μL of 1% (*w*/*v*) vanillin methanol and 500 μL of 25% (*v*/*v*) sulfuric acid methanol. The mixture was placed in a water bath at 30 °C for 15 min, and the absorbance was recorded at 500 nm using a UV-2600 spectrophotometer (Shimadzu Co., Kyoto, Japan). The standard curve was made by the epicatechin standard. The binding amount and loading efficiency of GSP can be expressed as Equations (1) and (2), respectively:

(1) $$\mathrm{Binding}\;\mathrm{amount}\;=\;\left(\mathrm{Total}\;\mathrm{contents}\;\mathrm{of}\;\mathrm{GSP}\left(\mathrm{mg}\right)\;–\;\mathrm{Free}\;\mathrm{content}\;\mathrm{of}\;\mathrm{GSP}\right)\;\left(\mathrm{mg}\right)/\mathrm{potato}\;\mathrm{starch}\;\left(g\right),$$

(2) $$\mathrm{Loading}\;\mathrm{efficiency}\;\left(\%\right)\;=\;\left(\mathrm{Total}\;\mathrm{content}\;\mathrm{of}\;\mathrm{GSP}\;–\;\mathrm{Free}\;\mathrm{content}\;\mathrm{of}\;\mathrm{GSP}\right)/\mathrm{Total}\;\mathrm{content}\;\mathrm{of}\;\mathrm{GSP}\;\times \;100,$$

where the total contents of GSP for each group represents the GSP weight before mixing with potato starch in each group.

### 3.4. X-ray Diffractometer Analysis

X-ray diffractometer (X’PERT PRO, Panalytical B.V., Netherlands) was applied to record XRD patterns of samples as well as GSP at a wavelength of 0.154 nm. A Cu-Kα radiation source (40 mA and 40 kV) was used according to a previous method [37]. XRD patterns were acquired with a step size of 2° min^−1^ over a scattering range (2θ) of 4–40°. Jade software (Version 6.5; Material data Inc., Livermore, CA, USA) was used to analyze the XRD patterns. Relative crystallinity (Xc) was calculated by Equation (3) [38].

(3) $$\mathrm{Xc}=\frac{\mathrm{Ac}}{\mathrm{Ac}+\mathrm{Aa}}\times 100,$$

where Ac represents for the areas of crystalline and Aa represents for amorphous phases on the XRD profiles.

### 3.5. Pasting Properties Analysis

Pasting properties of the samples were characterized by a rapid visco analyzer (RVA) (Anton Paar Instruments Inc., Graz, Austria), according to the manufacturer’s instructions. Briefly, 1.5 g of the sample (compensate for 14% moisture basis) was accurately weighed into a canister and mixed with 15 mL distilled water. The samples were held at 50 °C for 1 min, heated to 95 °C for 7.5 min, then held at 95 °C for 5 min before cooling to 50 °C for 7.5 min, and finally held at 50 °C for 2 min. Peak viscosity, trough viscosity, final viscosity, and other parameters including pasting temperature and peak time, breakdown, and setback were recorded.

### 3.6. Thermal Properties Analysis

Differential scanning calorimeter (Mettler Toledo International, Inc., Greifensee, Switzerland) was applied to investigate the thermal properties of the samples [21]. A 3-microgram sample was accurately weighed in an aluminum pan and 9 μL deionized water was mixed with the sample. The pans were sealed and equilibrated at room temperature for 12 h. Then, the pans were heated from 20 to 110 °C in 10 min with an empty counterpart as the reference. STARe (Version 16.0; Mettler Toledo International Inc., Greifensee, Switzerland) was used to calculate peak temperature (Tp), onset temperature (To), conclusion temperature (Tc), and gelatinization enthalpy (ΔH).

### 3.7. Fourier Transform Infrared Analysis

Fourier transform infrared spectrophotometer (AVA TAR370, Thermo Nicolet Corporation, Madison, WI, USA) was used to analyze the short-range ordered structure of the samples according to its introduction. Briefly, the samples were finely ground with KBr and pressed into a pellet. FT-IR patterns were acquired with a resolution ratio of 4 cm^−1^ for 32 scans over a wavelength range of 4000 to 400 cm^−1^. FT-IR Patterns were analyzed with Ominic (Version 6.2; Thermo Electron Corporation, Waltham, MA, USA).

### 3.8. Gel Texture Analysis

The pasted samples produced in Section 3.5 were first transferred to the molds and stored at 4 °C for three days. TA XT2i (Stable Micro System, Surrey, UK) was applied to analyze the texture profile and was equipped with a P/50 cylindrical probe. The testing parameters were as follows: Pre-test speed of 1.0 mm·s^−1^, test speed of 1.0 mm·s^−1^, post-test speed of 1.0 mm·s^−1^, trigger force of 5 g, compression depth of 50%. Hardness, springiness, cohesiveness, chewiness, and resilience were recorded and calculated by Exponent (Version 4.0; Stable Micro Systems, Surrey, UK).

### 3.9. In Vitro Digestion

The digestion of potato starch as well as the complexes were performed, according to a previous study [30]. Briefly, 1 g of sample was dispersed in 50 mL of 100 mmol/L sodium acetate buffer with 5 mmol/L CaCl_2_ in beaker flasks and the pH was adjusted to 5.2. The mixture was heated at 90 °C for 20 min with magnetic stirring for the full gelatinization of starch. Then, the fully cooked samples were incubated at 37 °C for 10 min. Three milliliters of enzyme solution (containing 120 U/mL α-amylase and 80 U/mL amyloglucosidase) was transferred to the beaker flasks and initiated the digestion. The beaker flasks were placed at 37 °C for 120 min with an oscillation frequency of 200 rpm. The simulated digestion supernatant (500 μL) was collected at 20 and 120 min, respectively. The supernatant was transferred into 2.5 mL of absolute ethanol immediately to inactivate the enzymes. Then, the solutions were centrifuged at 2000 g for 10 min and 100 μL supernatant was mixed with 500 μL of dual enzyme solution containing amyloglucosidase and invertase (32.5 U/mL, 112.5 U/mL, respectively) at 37 °C for 10 min to convert all the hydrolyzed starch into glucose [37]. A glucose oxidase/peroxidase assay kit was applied to measure the glucose concentrations according to the manufacturer’s instructions. Rapidly digestible starch (RDS), slowly digestible starch (SDS), and resistant starch (RS) were calculated by Equations (4)–(6), where 0.9 was the conversion coefficient from starch to glucose. G20 and G120 are the glucose content at 20 and 120 min, respectively, and TS represents total starch at the beginning.

(4) RDS =0.9 × G20,

(5) $$\mathrm{SDS}\;=0.9\;\times \;\left(G120\;–\;G20\right),$$

(6) $$\mathrm{RS}\;=\;0.9\;\times \left(\mathrm{TS}\;–\;G120\right),$$

### 3.10. Statistical Analysis

The analysis was performed in triplicate. One-way analysis of variance (ANOVA) in IBM SPSS statistic 23 was used to analyze the data. Tukey’s honestly significant difference were used to separate the means at a significant level of 0.05.

## 4. Conclusions

GSP has the potential to modify the physicochemical and nutritional properties of potato starch to produce GSP–potato starch complexes. The binding amount indicated a high affinity between GSP and potato starch. The interaction forces between GSP and potato starch might be noncovalent bonding, especially hydrogen bonding, which also retards retrogradation of gels made by complexes. The interaction in complexes could provide a more ordered structure, which implied a better heat and shear stability than the native starch. Furthermore, binding GSP reduced potato starch digestion during starch hydrolysis with a lower content of RDS and higher content of SDS and RS. Complexation between potato starch and GSP might offer opportunities to modify starch with a slowly digestive property. The complexes might be used directly as a bioactive starch or applied in novel functional starchy food processing. Moreover, utilizing the starch–proanthocyanidin complexes to control the release of proanthocyanidins seems promising and needs further study.

## Figures and Tables

**Table 1:** Amount of grape seed proanthocyanidins that reacted with potato starch in aqueous ethanol solutions.

Samples	Binding Amount (mg/g)	Loading Efficiency (%)
Starch + 1.0% GSP	7.36 ± 0.05	73.41 ± 0.63
Starch + 2.0% GSP	13.86 ± 0.06	69.19 ± 0.36
Starch + 3.0% GSP	20.57 ± 0.06	68.49 ± 0.13
Starch + 4.0% GSP	27.98 ± 0.04	69.91 ± 0.08
Starch + 4.5% GSP	32.20 ± 0.04	71.51. ± 0.05
Starch + 5.0% GSP	35.72 ± 0.02	71.40 ± 0.05

**Table 2:** Pasting parameters: pasting temperature (°C) and final viscosity (cP) for native potato starch and GSP-potato starch complexes.

Samples ^1^	Final Viscosity (cP)	Pasting Temperature (°C)
Native starch	1406.5 ± 24.75 ^f^	65.08 ± 0.04 ^f^
Starch + 0.0% GSP	4119.0 ± 49.50 ^a^	91.90 ± 0.64 ^e^
Starch + 1.0% GSP	3823.5 ± 105.36 ^b^	93.10 ± 0.57 ^c,d^
Starch + 2.0% GSP	3604.5 ± 71.42 ^b,c^	92.70 ± 0.71 ^d,e^
Starch + 3.0% GSP	3487.5 ± 84.15 ^c^	93.08 ± 0.53 ^c,d^
Starch + 4.0% GSP	3356.0 ± 155.56 ^c,d^	93.90 ± 0.57 ^b,c^
Starch + 4.5% GSP	3149.5 ± 183.14 ^d^	94.50 ± 0.28 ^a,b^
Starch + 5.0% GSP	2623.0 ± 93.34 ^e^	95.10 ± 0.03 ^a^

^1^ Different lowercase letters in the same column indicate a statistically significant difference (*p* < 0.05).

**Table 3:** Thermal properties of native potato starch and GSP–potato starch complexes.

Samples ^1^	To (°C)	Tp (°C)	Tc (°C)	ΔH (J/g)
Native starch	62.7 ± 0.7 ^a^	65.5 ± 0.3 ^a^	68.4 ± 1.0 ^a^	13.7 ± 0.3 ^a^
Starch + 0.0% GSP	52.0 ± 0.2 ^b^	58.6 ± 0.4 ^b^	64.6 ± 0.8 ^b^	0.9 ± 0.2 ^d^
Starch + 1.0% GSP	53.5 ± 0.9 ^b^	59.5 ± 0.7 ^b^	65.4 ± 1.5 ^b^	1.2 ± 0.6 ^b,c,d^
Starch + 2.0% GSP	53.6 ± 2.1 ^b^	60.1 ± 1.6 ^b^	65.2 ± 0.4 ^b^	0.9 ± 0.4 ^d^
Starch + 3.0% GSP	53.4 ± 2.8^b^	59.8 ± 0.2 ^b^	65.2 ± 1.3 ^b^	1.1 ± 0.2 ^c,d^
Starch + 4.0% GSP	54.8 ± 0.8 ^b^	59.4 ± 0.3 ^b^	65.5 ± 0.3 ^b^	1.3 ± 0.1 ^b,c,d^
Starch + 4.5% GSP	53.0 ± 1.4 ^b^	59.3 ± 0.8 ^b^	66.0 ± 0.9 ^b^	1.5 ± 0.1 ^b^
Starch + 5.0% GSP	53.2 ± 0.8 ^b^	59.7 ± 1.1 ^b^	66.1 ± 1.0 ^b^	1.5 ± 0.2 ^b,c^

^1^ Different lowercase letters in the same column indicate a statistically significant difference (*p* < 0.05).

**Table 4:** Texture profile analysis of native potato starch and GSP-potato starch complexes gel.

Samples ^1^	Hardness (g)	Springiness (mm)	Cohesiveness	Chewiness (g)	Resilience
Native starch	1290.007 ± 37.875 ^b^	0.874 ± 0.031 ^a^	0.83 ± 0.014 ^a^	936.999 ± 65.191 ^a^	0.426 ± 0.013 ^b^
Starch + 0.0% GSP	1392.628 ± 42.612 ^a^	0.909 ± 0.023 ^a,b^	0.811 ± 0.02 ^a^	1029.505 ± 82.358 ^a^	0.532 ± 0.023 ^a^
Starch + 1.0% GSP	1153.262 ± 35.037 ^c^	0.929 ± 0.02 ^a,b^	0.677 ± 0.022 ^b^	725.317 ± 33.782 ^b^	0.47 ± 0.013 ^c^
Starch + 2.0% GSP	947.100 ± 31.757 ^d^	0.862 ± 0.022 ^b^	0.601 ± 0.029 ^c^	491.446 ± 45.062 ^c^	0.32 ± 0.017 ^d^
Starch + 3.0% GSP	852.324 ± 26.475 ^e^	0.724 ± 0.021 ^c^	0.569 ± 0.018 ^c^	350.977 ± 10.008 ^d^	0.277 ± 0.021 ^e^
Starch + 4.0% GSP	816.614 ± 31.379 ^e,f^	0.644 ± 0.023 ^d^	0.551 ± 0.03 ^c^	289.698 ± 24.558 ^d,e^	0.201 ± 0.011 ^f^
Starch + 4.5% GSP	747.402 ± 13.089 ^g,h^	0.614 ± 0.011 ^d,e^	0.425 ± 0.021 ^d^	194.882 ± 8.587 ^e,f^	0.205 ± 0.017 ^f^
Starch + 5.0% GSP	719.548 ± 4.184 ^h^	0.580 ± 0.027 ^e^	0.394 ± 0.015 ^d^	164.122 ± 3.529 ^f^	0.185 ± 0.016 ^f^

^1^ Different lowercase letters in the same column indicate a statistically significant difference (*p* < 0.05).

## References

[1] Tian J., Chen S., Chen J., Liu D., Ye X. (2018). Cooking Methods Altered the Microstructure and Digestibility of the Potato. Starch.

[2] Food and Agricultural Organization. http://www.fao.org/faostat/en/#data.

[3] Ek K.L., Brand-Miller J., Copeland L. (2012). Glycemic effect of potatoes. Food Chem..

[4] Schwingshackl L., Hoffmann G. (2013). Long-term effects of low glycemic index/load vs. high glycemic index/load diets on parameters of obesity and obesity-associated risks: A systematic review and meta-analysis. Nutr. Metab. Cardiovasc. Dis..

[5] Oladele A.K., Duodu K.G., Emmambux N.M. (2019). Pasting, flow, thermal and molecular properties of maize starch modified with crude phenolic extracts from grape pomace and sorghum bran under alkaline conditions. Food Chem..

[6] Ping L., Pizzi A., Guo Z.D., Brosse N. (2012). Condensed tannins from grape pomace: Characterization by FTIR and MALDI TOF and production of environment friendly wood adhesive. Ind. Crop. Prod..

[7] Wang M., Jiang J., Tian J., Chen S., Ye X., Hu Y., Chen J. (2019). Inhibitory mechanism of novel allosteric inhibitor, Chinese bayberry (*Myrica rubra* Sieb. et Zucc.) leaves proanthocyanidins against α-glucosidase. J. Funct. Foods.

[8] Amoako D.B., Awika J.M. (2019). Resistant starch formation through intrahelical V-complexes between polymeric proanthocyanidins and amylose. Food Chem..

[9] Guo Y., Yan M., Lin S., Yang X., Fu C., Huang D. (2017). Binding Interaction of Selected Proanthocyanidins Possessing Hypoglycemic Activity with Common Food Raw Materials. Food Sci..

[10] Sun L., Miao M. (2019). Dietary polyphenols modulate starch digestion and glycaemic level: A review. Crit. Rev. Food Sci. Nutr..

[11] Barrett A.H., Farhadi N.F., Smith T.J. (2018). Slowing starch digestion and inhibiting digestive enzyme activity using plant flavanols/tannins—A review of efficacy and mechanisms. LWT.

[12] Zhu F. (2015). Interactions between starch and phenolic compound. Trends Food Sci. Technol..

[13] Yang L., Zhang B., Yi J., Liang J., Liu Y., Zhang L.M. (2013). Preparation, characterization, and properties of amylose-ibuprofen inclusion complexes. Starch.

[14] Van Hung P., Phat N.H., Phi N.T.L. (2013). Physicochemical properties and antioxidant capacity of debranched starch–ferulic acid complexes. Starch.

[15] Star A., Steuerman D.W., Heath J.R., Stoddart J.F. (2002). Starched Carbon Nanotubes. Angew. Chem. Int. Ed..

[16] Obiro W.C., Sinha Ray S., Emmambux M.N. (2012). V-amylose Structural Characteristics, Methods of Preparation, Significance, and Potential Applications. Food Rev. Int..

[17] Tian J., Ogawa Y., Shi J., Chen S., Zhang H., Liu D., Ye X. (2019). The microstructure of starchy food modulates its digestibility. Crit. Rev. Food Sci..

[18] Chi C., Li X., Feng T., Zeng X., Chen L., Li L. (2018). Improvement in Nutritional Attributes of Rice Starch with Dodecyl Gallate Complexation: A Molecular Dynamic Simulation and in Vitro Study. J. Agric. Food Chem..

[19] Tian J., Chen S., Zhang H., Fang H., Sun Y., Liu D., Linhart R.J., Ye X. (2018). Existing cell wall fragments modify the thermal properties and hydrolysis of potato starch. Food Hydrocolloids.

[20] Liu J., Wang M., Peng S., Zhang G. (2011). Effect of Green Tea Catechins on the Postprandial Glycemic Response to Starches Differing in Amylose Content. J. Agric. Food Chem..

[21] Wang M., Shen Q., Hu L., Hu Y., Ye X., Liu D., Chen J. (2018). Physicochemical properties, structure and in vitro digestibility on complex of starch with lotus (*Nelumbo nucifera* Gaertn.) leaf flavonoids. Food Hydrocolloids.

[22] Wang S., Wang J., Wang S., Wang S. (2017). Annealing improves paste viscosity and stability of starch. Food Hydrocolloids.

[23] Stute R. (1992). Hydrothermal Modification of Starches: The Difference between Annealing and Heat/Moisture -Treatment. Starch.

[24] Chai Y., Wang M., Zhang G. (2013). Interaction between Amylose and Tea Polyphenols Modulates the Postprandial Glycemic Response to High-Amylose Maize Starch. J. Agric. Food Chem..

[25] Hu X., Xu X., Jin Z., Tian Y., Bai Y., Xie Z. (2011). Retrogradation properties of rice starch gelatinized by heat and high hydrostatic pressure (HHP). J. Food Eng..

[26] Cardoso M.B., Putaux J., Samios D., Da Silveira N.P. (2007). Influence of alkali concentration on the deproteinization and/or gelatinization of rice starch. Carbohyd. Polym..

[27] Zhou X., Baik B., Wang R., Lim S. (2010). Retrogradation of waxy and normal corn starch gels by temperature cycling. J. Cereal Sci..

[28] Kizil R., Irudayaraj J., Seetharaman K. (2002). Characterization of Irradiated Starches by Using FT-Raman and FTIR Spectroscopy. J. Agric. Food Chem..

[29] Mathew S., Abraham T.E. (2007). Physico-chemical characterization of starch ferulates of different degrees of substitution. Food Chem..

[30] Englyst H.N., Kingman S.M., Cummings J.H. (1992). Classification and measurement of nutritionally important starch fractions. Eur. J. Clin. Nutr..

[31] Martínez P., Peña F., Bello-Pérez L.A., Núñez-Santiago C., Yee-Madeira H., Velezmoro C. (2019). Physicochemical, functional and morphological characterization of starches isolated from three native potatoes of the Andean region. Food Chem..

[32] Dupuis J.H., Liu Q. (2019). Potato Starch: A Review of Physicochemical, Functional and Nutritional Properties. Am. J. Potato Res..

[33] Yilmazer-Musa M., Griffith A.M., Michels A.J., Schneider E., Frei B. (2012). Grape Seed and Tea Extracts and Catechin 3-Gallates Are Potent Inhibitors of alpha-Amylase and alpha-Glucosidase Activity. J. Agric. Food Chem..

[34] Barros F., Awika J., Rooney L.W. (2014). Effect of molecular weight profile of sorghum proanthocyanidins on resistant starch formation. J. Sci. Food Agric..

[35] Zheng Y., Tian J., Kong X., Yang W., Yin X., Xu E., Chen S., Liu D., Ye X. (2020). Physicochemical and digestibility characterisation of maize starch–caffeic acid complexes. LWT.

[36] Sun B., Ricardo-da-Silva J.M., Spranger I. (1998). Critical Factors of Vanillin Assay for Catechins and Proanthocyanidins. J. Agric. Food Chem..

[37] Tian J., Cai Y., Qin W., Matsushita Y., Ye X., Ogawa Y. (2018). Parboiling reduced the crystallinity and in vitro digestibility of non-waxy short grain rice. Food Chem..

[38] Nara S., Komiya T. (1983). Studies on the Relationship Between Water-satured State and Crystallinity by the Diffraction Method for Moistened Potato Starch. Starch.

